# Curved Nanofiber Network Induces Cellular Bridge Formation to Promote Stem Cell Mechanotransduction

**DOI:** 10.1002/advs.202204479

**Published:** 2022-11-16

**Authors:** Qian Sun, Fang Pei, Man Zhang, Bo Zhang, Ying Jin, Zhihe Zhao, Qiang Wei

**Affiliations:** ^1^ Department of Orthodontics State Key Laboratory of Oral Diseases National Clinical Research Center for Oral Diseases West China Hospital of Stomatology Sichuan University Chengdu 610041 P. R. China; ^2^ College of Polymer Science and Engineering State Key Laboratory of Polymer Materials and Engineering Sichuan University Chengdu 610065 P. R. China; ^3^ College of Biomedical Engineering Sichuan University Chengdu 610065 P. R. China

**Keywords:** cell differentiation, curved nanofibers, extracellular matrix, mechanotransduction, stem cells

## Abstract

Remarkable exertions are directed to reveal and understand topographic cues that induce cell mechanical sensitive responses including lineage determination. Extracellular matrix (ECM) is the sophisticated ensemble of diverse factors offering the complicated cellular microenvironment to regulate cell behaviors. However, the functions of only a few of these factors are revealed; most of them are still poorly understood. Herein, the focus is on understanding the curved structure in ECM network for regulating stem cell mechanotransduction. A curved nanofiber network mimicking the curved structure in ECM is fabricated by an improved electrospinning technology. Compared with the straight fibers, the curved fibers promote cell bridge formation because of the cytoskeleton tension. The actomyosin filaments are condensed near the curved edge of the non‐adhesive bridge in the bridging cells, which generates higher myosin‐II‐based intracellular force. This force drives cell lineage commitment toward osteogenic differentiation. This study enriches and perfects the knowledge of the effects of topographic cues on cell behaviors and guides the development of novel biomaterials.

## Graphical abstract

The curved nanofibers in cell adhesive substrates promote the formation of cell bridges. The actomyosin stress fibers are condensed in the overhanging regions of the edge of the bridging cells. The stiff stress fibers not only exhibit high tension against cell edge deformation to maintain the stability of cell bridges but also generate traction force to activate the mechanotransduction pathways.

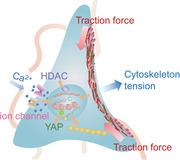

## 1 Introduction

The current studies realize both physical and biochemical factors acting on cell physiological and behavioral functions. Besides traditional chemical effects, novel biomaterials have been developed by mimicking the extracellular matrix (ECM) stiffness,^[^ ^1^ ^]^ stress relaxation,^[^ ^2^ ^]^ degradation,^[^ ^3^, ^4^ ^]^ roughness,^[^ ^5^, ^6^ ^]^ alignment,^[^ ^7^ ^]^ ligand diffusion,^[^ ^8^ ^]^ ligand spacing,^[^ ^9^, ^10^ ^]^ and other physical properties,^[^ ^11^, ^12^ ^]^ which influence various cell behaviors and functions through cellular mechanosensing and mechanotransduction.^[^ ^13^ ^]^ In general, cellular mechanosensing and mechanotransduction are mediated by intracellular traction force, which is balanced and determined by the physical cues in cell microenvironment.^[^ ^14^ ^]^ Actomyosin generates and transmits the traction force to both adhesome and nucleus.^[^ ^14^ ^]^ The adhesomes consist of integrin clusters and structural proteins, which undergo conformational changes under intracellular force to initiate multiple signaling cascades.^[^ ^14^, ^15^ ^]^ The force transmission toward cell nucleus remodels the chromatin to mediate the transcriptional activity for altering the cell phenotypes.^[^ ^16^, ^17^ ^]^

**Figure 1:**
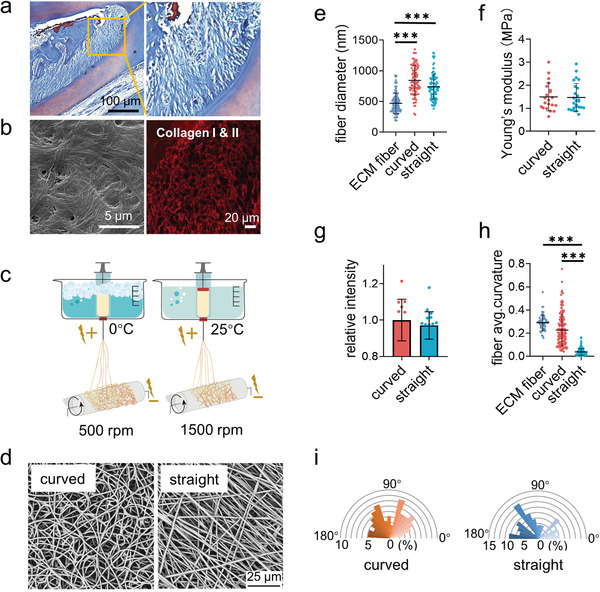
Fabrication and characterization of the curved and straight nanofiber network. a) The Representative images of masson staining of the periodontal tissues. b) The SEM (left) image of the decellularized periodontal ligament tissues and the representative fluorescence image of the collagen I and II (right) in periodontal tissues. c) Scheme of the curved and straight nanofiber network fabrication. The curved and straight fiber network require 0 °C and 25 °C electrospinning temperature, respectively. d) The representative SEM images of the curved and straight fibers (three technical replicates). e) The diameter of the ECM fibers in the periodontal tissues and the artificial fibers (*n* = 100, two technical replicates). f) Young's modulus of the curved and straight nanofiber network as detected by Nanoindenter (*n* = 20, two technical replicates). g) Specific surface area of the curved and straight surfaces as detached by the fluorescent intensity of the adsorbed FITC‐BSA at 562 nm (*n* = 12, two technical replicates). h) The average curvature of the ECM fibers in the periodontal tissues and the artificial fibers (*n* = 160, two technical replicates). i) The orientation angles (*n* = 100, two technical replicates) of the curved and straight fibers.

Topographic feature is one of the most common physical cues in cell microenvironment because the ECM is composed of fibrous structures.^[^ ^18^, ^19^ ^]^ The composition and the topology of the fibers undertake the physical/mechanical properties of the environmental stiffness, strength, extensibility, and tissue resilience.^[^ ^20^, ^21^ ^]^ Numerous biomaterials with fibrous structure have been designed^[^ ^22^ ^]^ benefiting from the developing technologies for fiber synthesis and preparation, such as electrospinning,^[^ ^23^ ^]^ freeze casting,^[^ ^24^ ^]^ 3D bio‐printing,^[^ ^25^ ^]^ and others. Among these, electrospinning is the most frequently utilized because of its high efficiency and convenience for fabricating nano‐size fibers. However, great progress has been made in manufacturing scaffolds that only match the straight topology of the natural ECM fibers. In fact, there are also curved structures in native ECM network, which could absorb more strain energy than straight fibers to buffer the mechanical load generated by the attached skin, muscle, and bone.^[^ ^26^, ^27^, ^28^ ^]^ The curved collagen fiber in ECM was reported to be unbent by cancer cells during tumor development.^[^ ^29^ ^]^ The periodontal ligament, a fibrous connective tissue, was detected to contain curved structures as well by ourselves (**Figure** **1**a,b). The function of such structure at the cellular level is yet to be understood. The lack of methods for fabricating curved nanofiber networks also limits the related studies.

To address these questions, we introduce a technology of cryogenic electrospinning to fabricate the curved nanofiber membranes as the promising ECM mimicking biomaterials as well as the tool to study cell response to curved structures. Surprisingly, the curved fibers alter stem cell adhesive behaviors compared to the straight fibers. The cells just adhere along the straight fibers but cross the curved fibers to form cell bridges (indicating cell bodies overhung rather than attached on fibers). The bridge formation rearranges the actomyosin cytoskeleton distribution to obtain the extra intracellular force, as explained by a 2D Laplace's law model, which enhances cell mechanotransduction and promotes osteogenic differentiation. Our new finding and understanding of these biomechanical principles are critical for promoting the development of tissue engineering.

## 2 Results and Discussion

### 2.1 Constructing Curved Nanofiber Network Via Cryogenic Electrospinning

The electrospinning technique is the most versatile method to fabricate the nanofiber scaffolds for tissue engineering at present as the fibrous structures in natural ECM are in nano‐size.^[^ ^30^ ^]^ It uses an electric field to overcome the surface tension of a polymer solution to collect randomly oriented fibers and form a nanofiber membrane.^[^ ^31^ ^]^ The parameters, such as voltage, flow rate, distance to the collector, polymer solution concentration, solution conductivity, solvent, humidity, and temperature, determine the characteristics of the fiber nonlinearly.^[^ ^31^ ^]^ We devise a low‐temperature model system for fabricating the curved polycaprolactone (PCL) nanofiber membrane. For straight nanofiber matrix, the rotating receiver speed and temperature are 1500 rpm and 25 °C. For curved nanofiber matrix, the rotating receiver speed and temperature are decreased to 400 rpm and 0 °C (Figure 1c). It is clear to observe that the electrospinning PCL nanofibers under different fabrication conditions display distinct directions as shown by the scanning electron microscopy (SEM) images in Figure 1d. Each nanofiber follows one direction from the beginning to the end and forms a strong network in the “straight” matrix. Meanwhile, the “curved” fibers adjust the direction of extension continuously and intertwine with each other.

The ECM fibers in tissue have the broadly distributed diameter from 50 nm to 10 um as reported in literatures^[^ ^28^, ^32^, ^33^ ^]^ and confirmed in Figure 1a,b, as the periodontal tissues contained both small fibers and large bundles. The average diameter of the electrospinning curved and straight nanofibers was ≈800 nm, which was in the range of the natural fibers and was consistent with the size of the fibers observed in the periodontal tissues (Figure 1e). The size of the fibers was enough for cells to form large adhesion clusters, for example, focal adhesion along the direction of the nanofibers.^[^ ^7^, ^34^ ^]^ The Young's modulus of the straight and curved nanofiber matrixes was ≈1.5 MPa as measured by nanoindenter (Figure 1f), which was rigid enough to support the highest cellular traction force.^[^ ^35^ ^]^ The curved and straight nanofiber matrixes have the similar specific surface area, which was indicated by the fluorophore labelled bovine serum albumin (BSA) (Figure 1g; Figure S1, Supporting Information). The direction of both nanofibers also exhibited the similar isotropy (Figure 1i). In order to describe the morphological difference between these two types of fibers, we used an edge curvature method (Kappa) for analysis.^[^ ^36^ ^]^ The curved nanofiber network displayed a significantly higher level of curvature in keeping with the native ECM fibrous structures compared to the straight nanofiber network (Figure 1h).^[^ ^27^, ^28^ ^]^ PCL has good biocompatibility, and the PCL nanofibers prepared by electrospinning have the same chemical structure, which ensures that the physical factors can be modulated independent of the chemical factors. In addition, the ligand type can affect cell adhesion and phenotype determination.^[^ ^12^, ^37^ ^]^, The PCL non‐specifically adsorbs the ECM proteins expressed by cells; thus, the ligands would be similar to the natural ECM.^[^ ^38^ ^]^ Therefore, we had successfully established the model surfaces with straight and curved features to study cellular behaviors.

### 2.2 Curved Fiber Promotes Cell Proliferation and Osteogenic Differentiation

**Figure 2:**
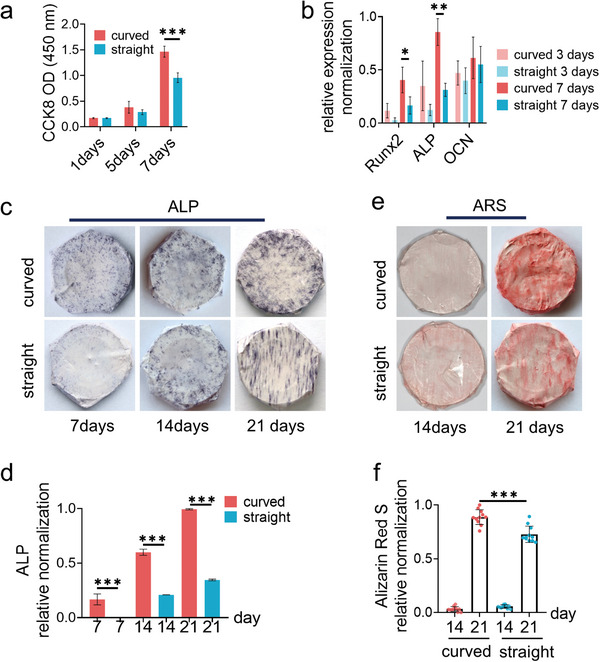
The PDLSC phenotypes on the structural fibers. a) The cell viability as assessed by using the CCK8 assay (*n* = 9, two biological replicates). b) The mRNA expression levels of the osteogenic genes RUNX2 (early marker), ALP (middle marker), and OCN (late marker) as analyzed by RT‐qPCR after cell culture for 3 and 7 days, respectively. c) The representative images and d) the related quantification data of ALP activity at 7, 14, and 21 days after cell culture (*n* = 4, three biological replicates). e) The representative images and f) the related quantification data of Alizarin red S staining in osteoblasts at 14 and 21 days after cell culture (*n* = 4, three biological replicates).

The periodontal ligament stem cells (PDLSCs) were isolated to evaluate the effect of fiber structures on cell proliferation and differentiation, the essential cell functions for odontogenesis. As indicated by the CCK8 assay (**Figure** **2**a), the curved nanofiber network improved cell proliferation significantly comparing with the straight nanofiber network after 7 days of cell culture. The osteogenic markers runt‐related transcription factor 2 (RUNX2, early marker), alkaline phosphatase (ALP, intermediate marker), and osteocalcin (OCN, late marker) were evaluated for cell osteogenic differentiation. The gene expression of these markers at 3 days and 7 days were analyzed by the reverse transcription‐quantitative polymerase chain reaction (RT‐qPCR), as shown in Figure 2b. The ALP protein activity was also recorded at 7, 14, and 21 days (Figure 2c,d). The calcium deposition, the mark of the matured osteogenesis, was detected by Alizarin Red S (ARS) staining at 21 days (Figure 2e,f). All these results indicated that cells exhibited stronger osteogenic differentiation on the curved nanofibers comparing with straight fibers.

### 2.3 Curved Fiber Enhances Intracellular Force and Cell Mechanosensing

**Figure 3:**
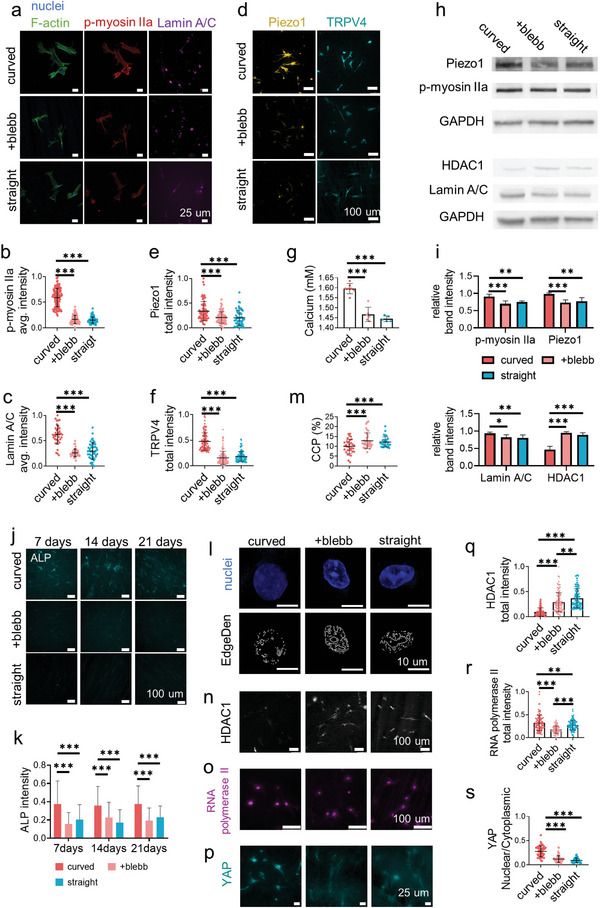
The PDLSC mechanotransduction on the structural fibers. a) The representative fluorescence images of nucleus (blue), filamentous actin (F‐actin, green), phospho‐myosin IIa (Ser1943) (red), and Lamin A/C (magenta) staining after 24 h of cell culture on the curved and straight fibers or on the curved fibers with blebbistatin (blebb) treatment. b) The average fluorescence intensity of p‐myosin IIa (*n* = 50, two technical replicates, two biological replicates) and c) Lamin A/C (*n* = 35, two technical replicates, two biological replicates) on the related surfaces. d) The representative fluorescence images of Piezo1 (gold) and TRPV4 (cyan) of the cell culture on the related surfaces. The total fluorescence intensity per cell of e) Piezo1 and f) TRPV4 (*n* = 100, two technical replicates, three biological replicates) on the related surfaces. g) The relative concentration of calcium ion on the related surfaces (*n* = 5, five biological replicates, two technical replicates). h,i) Western blot analysis of Piezo1, phospho‐myosin IIa (Ser1943), HDAC1, and Lamin A/C after 24 h of cell culture on the curved and straight fibers or on the curved fibers with blebbistatin (blebb) treatment. j) The representative fluorescence images and k) the related quantification of the ALP for 7, 14, and 21 days after the cells cultured on the related surface (*n* = 50, two biological replicates). l) The representative fluorescence images of the single nucleus (DAPI staining, blue) and the Sobel edge detection (gray, *n* = 30, two technical replicates, two biological replicates) on the related surfaces. m) Analysis of chromatin condensation parameter (CCP) measured from stained nuclei on the related surface. n) The representative fluorescence images of HDAC1 (gray) and o) RNA polymerase II (magenta) staining after 24 h of cell culture on the related surfaces (*n* = 100, two technical replicates, two biological replicates). p) Representative fluorescence images of PDLSC stained with anti‐YAP (cyan) on the curved and straight fibers or on the curved fibers with blebbistatin (blebb) treatment. q) HDAC1 (*n* = 100, two technical replicates, two biological replicates), r) RNA polymerase II total intensity (*n* = 100, two technical replicates, two biological replicates) and s) the ratio of total nuclear intensity to total cytoplasmic intensity of YAP (*n* = 50, two technical replicates, two biological replicates) on the related surface.

As both of the cell proliferation and osteogenic differentiation capacities are the downstream functions of the cell adhesion and mechanotransduction, we thus focused on cell mechanosensing. The adhesion state and the cell mechanotransduction were analyzed after 24 h of cell culture. As shown in **Figure** **3**a; Figure S2, Supporting Information, the adhesion area of the cells on different fibers was similar. The cell spread area could mirror the intracellular force on flat surfaces,^[^ ^1^ ^]^ but the correlation was interfered on the patterned surfaces.^[^ ^7^ ^]^ Therefore, the mechanosensing was investigated in deep. Non‐muscle myosin II generated intracellular force and mediated the changes in cytoskeleton construction.^[^ ^1^ ^]^ The myosin II activity was identified by the immunofluorescence and western blot assay of the phosphorylated myosin IIa (p‐myosin IIa) at Ser1943 (Figure 3a,b,h,i). The cells on the curved fibers showed higher p‐myosin IIa fluorescence intensity than on the straight fibers. Calcium ions were involved in nearly all of the mechanosensing and mechanotransduction processes.^[^ ^39^ ^]^ The Piezo1 and TRPV4 ion channels were responsible to identify the mechanical stimulation.^[^ ^40^, ^41^ ^]^ The upregulated expressions of Piezo1 and TRPV4 were observed on curved fibers, which triggered calcium enrichment (Figure 3d–i).

The intracellular force transmitted the matrix topographic cues through actomyosin and finally entered the nucleus, in which the gene transcription took place.^[^ ^14^ ^]^ Recent evidence shows that one of the main nuclear structural proteins, Lamin A/C, changes its construction in response to the environmental physical factors and contributes to the lineage commitment.^[^ ^17^ ^]^ The intensity of Lamin A/C on the curved fibers was in a higher level comparing with that on the straight fibers (Figure 3a,c,h,i). These results suggested that the curved fibers promoted cell mechanosensing and the topographic cues could be delivered into the nucleus. To further confirm the intracellular force and cell mechanosensing playing the leading role in guiding cell lineage determination on the structural networks, the blebbistatin, a small molecular inhibitor to decrease the myosin II activity, was utilized to treat the cells. With the inhibitor treatment on the curved fibers, the phosphorylation level of myosin IIa in cells decreased to the same level as on the straight fibers (Figure 3a,b,h,i). The blebbistatin treatment also reduced the activity of the Piezo1 and TRPV4 channels as well as the calcium concentration to the same level as on the straight fiber (Figure 3d–i). The Lamin A/C level decreased as well after myosin II inhibition (Figure 3a,c,h,i) indicating the efficient disruption of the intracellular force transmission.

As indicated by the ALP activity from 7 to 21 days (Figure 3j,k), the intracellular force inhibition erased the increased osteogenic differentiation level induced by the fiber curvature. It validated that the fiber structures regulated cell lineage determination through cell mechanosensing and mechanotransduction, which were promoted by the curved structure.

The cytoskeleton transmitted mechanical force to Lamin A/C, which further stretched the condensed chromatin to open the target genes.^[^ ^17^, ^42^ ^]^ As shown in Figure 3l,m, the enhanced force on curved fibers induced chromatin unfolding in nucleus based on the different edge density assay.^[^ ^43^ ^]^ In chromatin, histone was tightly binding to DNA and adjusted the chromatin accessibility. The acetylation status of histone was dynamic and mechanosensitive, balanced by the proceedings of histone acetyltransferases (HATs) and histone deacetylases (HDACs), which could open or close the specific genes.^[^ ^44^ ^]^ The quantitative analysis indicated the HDAC1 activity in the cells on the curved fibers was much lower than that on the straight fibers or the cells treated with blebbistatin on the curved fibers (Figure 3h,i,n,q). The transcription of protein‐coding genes was further carried out on the accessible chromatin by the enzyme RNA polymerase II and other co‐regulators.^[^ ^45^ ^]^ The cells on the curved fibers exhibited a significant increase of RNA polymerase II, which was in accordance with HDAC1 activity (Figure 3o,r). On the other hand (Figure 3p,s), the cell force on the curved fibers successfully promoted the nuclear translocation of Yes‐associated protein (YAP), which was recognized as the force‐sensitive transcriptional coregulator. Therefore, the curved fiber activated intracellular force and mechanotransduction to remodel chromatin and activate transcriptional regulators for altering fate determination.

### 2.4 Cells Bridge the Curved Fibers to Acquire the Cellular Tension

**Figure 4:**
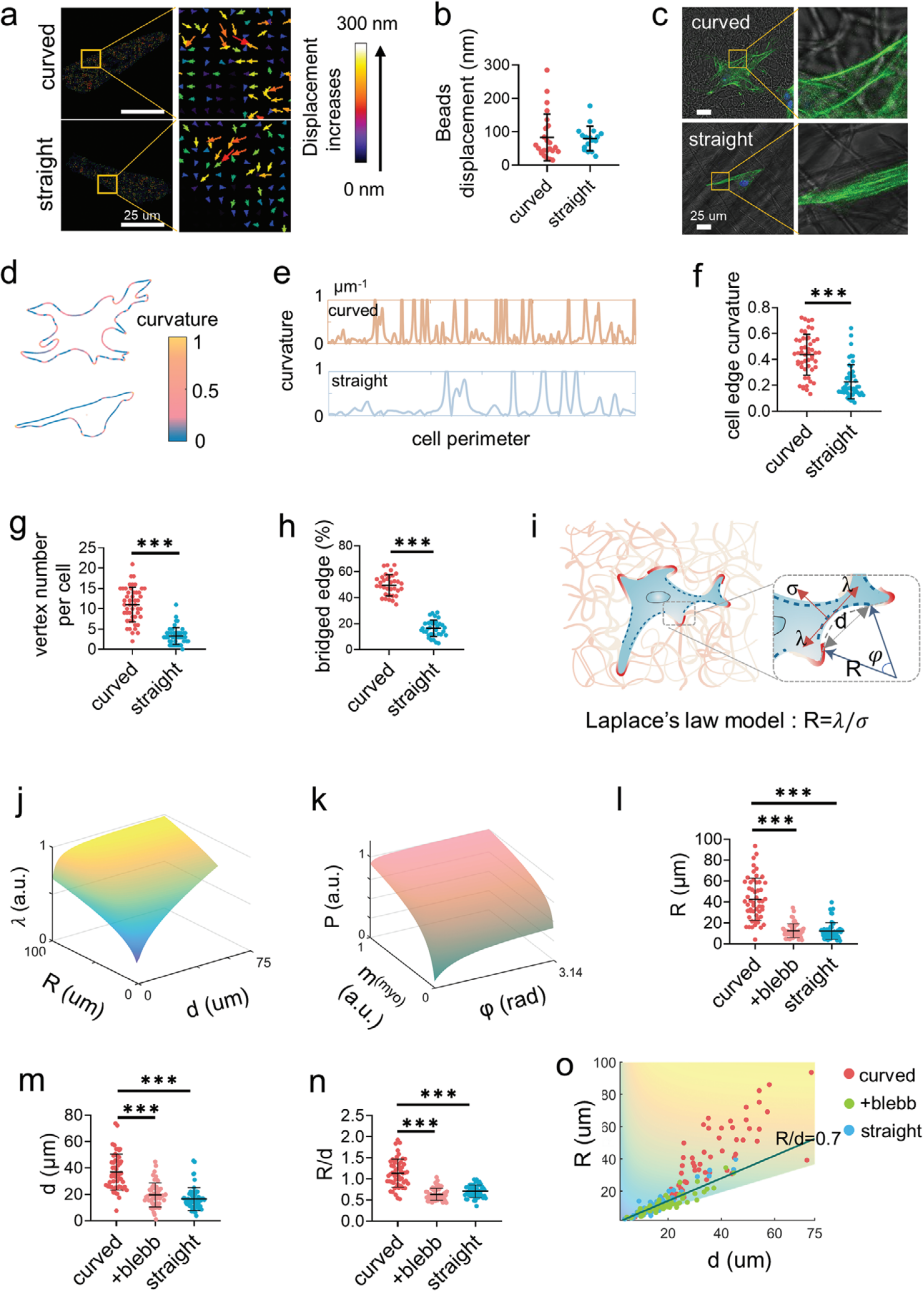
Cells bridge on the curved fibers. a) The representative displacement fields and b) the displacement quantification of the deformation of the curved and straight fibers under cell traction force as indicated by the embedded fluorescent microbeads (*n* = 50, two technical replicates). c) The representative images of the F‐actin labelled PDLSCs on the curved and straight fibers. d) Analysis of the edge curvature of the representative cells from (c) with *MATLAB* analysis. e) The plotting of the curvature fluctuation of the complete edge of the representative cells from (c) as analyzed by MATLAB. The curvature greater than 1 was not necessary to be shown. f) The average curvature (*n* = 50, two technical replicates), g) the vertex number (*n* = 50, two technical replicates), and h) the percentage of the bridged edge (*n* = 30, two technical replicates) of the single cells cultured on the curved and straight fibers. The point with curvature greater than 1 is defined as a vertex. i) Schematic of the 2D Laplace's law model adapts to cell non‐adhesive bridge. The radius of the curvature of the non‐adhesive bridge reflects the balance between the surface tension *σ* and the linear cell edge tension *λ* following *R* = *λ*/*σ*. The greater the *R*, the greater the force at the adhesion point. j) Model predicts the actomyosin traction force as a function of radius (*R*) and distance (*d*) as well as k) the effective pressure exerted by contractile bundle as a function of myosin motor density (*m*_(myo)_) and angle (*φ*) at the non‐adhesive bridge. Larger *R* and *d* lead to the increased traction force, while myosin aggregation contributes to intracellular force. l–n) Quantification of the radius of curvature (*R*), the distance between the two ends of the arc (*d*) (*n* = 20, two technical replicates), and the *R*/*d* value of the cells on the curved and straight fibers as well as the cells treated by blebbistatin on the curved fibers. o) Measurements of cellular bridge *R* and *d* over a range of linear cell edge tension (*λ*) consistent with model predictions. Decreased cellular traction force exhibited lower *R* and *d* (+blebb represents the cells treated by blebbistatin on the curved fibers).

The key question now is how the curved fibers enhance the intracellular force. We first examined the matrix deformation at the cell adhesion zone as the soft fibers and structures can be deformed during cell adhesion to apply extra elastic force to cells.^[^ ^5^, ^46^ ^]^ To track this minor deformation, the fluorescent nanobeads were homogeneously mixed into PCL solutions before electrospinning for fabricating the labeled nanofibers. The fiber deformation was recorded by imaging the displacement of the fluorophore‐labeled nanobeads during the process of cell adhesion. However, the PCL fibers were too rigid to be obviously deformed by the cells (**Figure** **4**a,b), so, the curved fibers would not apply extra elastic force to alter the cell mechanosensing.

To reveal the mechanism of cell mechanosensing on the curved fibers, we analyzed the geometric morphology of the spread cells in detail, despite there being no obvious difference in cell spread area on these two fiber structures as shown above. The stained cells with the merged bright‐field images of the fibers exhibited more information about the cell morphology and the relative position between the cells and the fibers (Figure 4c). It can be observed that the cells on the curved fibers exhibited distinct morphology from the cells on the straight fibers. The vertex structure of each individual cell was counted by defining the edge curvature exceeding 1, that is, the sharp edge, as the vertex (Figure 4d,e). The average vertex number per cell reached more than 10 on the curved fibers, while it was lower than 5 on the straight fibers (Figure 4g). The cells stably adhered at the vertex points, and the intracellular traction stretched the neighbor plasma membrane inward forming the sharp edges. The actomyosin‐based adhesive force was generated against the increase in the length of the cell contour line between each two vertices.^[^ ^47^ ^]^ Therefore, a higher vertex number indicated more adhesion sites and stronger intracellular traction force.

The cell edges were further analyzed to explore the vertex formation. In fact, the cells differed not only in geometric morphology but also in the way how they contacted the nanofibers. Most of the cell edges coincided with the straight fiber but not with the curved fibers. The cell edge could be divided into “contact region” and “non‐adhesive bridge” depending on the contact pattern between cell edges and surrounding nanofibers. Less edge region of the cells attached on the curved fibers comparing with the straight fibers (Figure 4h). The cell edge extension direction and the fiber elongation direction shared a common tangential line at the adhesive region but then diverged from each other due to the fiber curvature. The bridged edges became bent due to the lack of adhesive support. In contrast, the cells spread along the straight fibers forming continuous contact lines with more straight edges. The curvature of the cell edges on the curved fibers was significantly larger than that on the straight fibers (Figure 4c–f). The straight fibers formed border lines to determine the geometric morphology of the adhesive cells, while the curved fibers promoted cell bridging to bend their edges for vertex formation.

The 2D Laplace's law model was invoked to depict the effects of cell geometric morphology on the tension transmitted from the fibrous substrates to the cells (Figure 4i). *R*, the radius of the non‐adhesive bridges of the cell edges, reflects the linear tension (*λ*) related to the arc of the bridge area. The membrane tension (*σ*) follows *R* = *λ*/*σ*. Meanwhile, *d*, the distance between the two ends of the arc of the bridges is also used to characterize the magnitude of the tension. The *R*/*d* ratio reflects the intracellular tension.^[^ ^48^ ^]^ On the basis of this model, we identified the non‐adhesive bridges in the cell edge region and explored the cellular tension exerted in these regions (Figure 4c,d). The radius (*R*) and angle (*φ*) represented the length of the actomyosin bridge (*L*) and the distance (*d*) between the two ends of the arc as follows, $L=\varphi R,\;d=2R\sin(\frac{\varphi }{2})$. We independently varied and redefined each parameter in 2D Laplace's law model to illustrate the bridge sensitivity with regard to each parameter fluctuation.^[^ ^49^ ^]^ Together, the length of the actomyosin stress fiber bridge (*L*), the myosin motor density (*m*_(myo)_) which represents the overall myosin activity,^[^ ^47^ ^]^ the resistance for stress fiber displacements (*ζ*), and traction force of a single myosin motor (*k*) defined the growing function of actomyosin traction force. The 1D traction force along stress fiber is $\lambda =\frac{km_{\left(\mathrm{myo}\right)}L}{L+\zeta }$.^[^ ^49^ ^]^ Together with Laplace's low formula, *R* = *λ*/*σ*, predicts that $\lambda =km_{\left(myo\right)}\frac{1}{1+\zeta {\left(2R\arcsin(\frac{d}{2R})\right)}^{-1}}$. According to 2D Laplace's law model, the pressure (*P*) from bridge traction force on the cellular membrane is $P=\frac{km_{\left(\mathrm{myo}\right)}}{R}\;\frac{L}{L+\zeta }$.^[^ ^49^ ^]^ So far, we varied each parameter within the range of practical application and compared it with actual cell phenotype to plot traction distribution (Figure 4j,k). The *R* value of the bridged cell edges ranged from 4 to 93 µm on the curved fibers and from 4 to 35 µm on the straight fibers, respectively (Figure 4l; Figure S3, Supporting Information). Although the *d* value on the curved fibers was slightly larger (Figure 4m), the average *R*/*d* ratio was still obviously larger on the curved fibers (≈1.13) than on the straight fibers (≈0.72) (Figure 4n). This raised an interesting question: does the cell bridge tend to keep stable when the arc length and curvature radius fluctuate? Erdem et al. proposed the *R*/*d* values of ≈0.71 as the theoretical lower limit for bridge formation.^[^ ^48^ ^]^ Below this value, such non‐adhesive bridges collapse. In our case, the value of the cells attached to the straight fibers or treated with blebbistatin on curved fibers approached to 0.71 (Figure 4o). The experimental and simulation results were overall consistent. These results indicated the strong intracellular tension induced by bridge formation on the curved fibers and supported the vertex number analysis as shown above.

### 2.5 Actomyosin Cytoskeleton Induces Cell Bridge Formation

**Figure 5:**
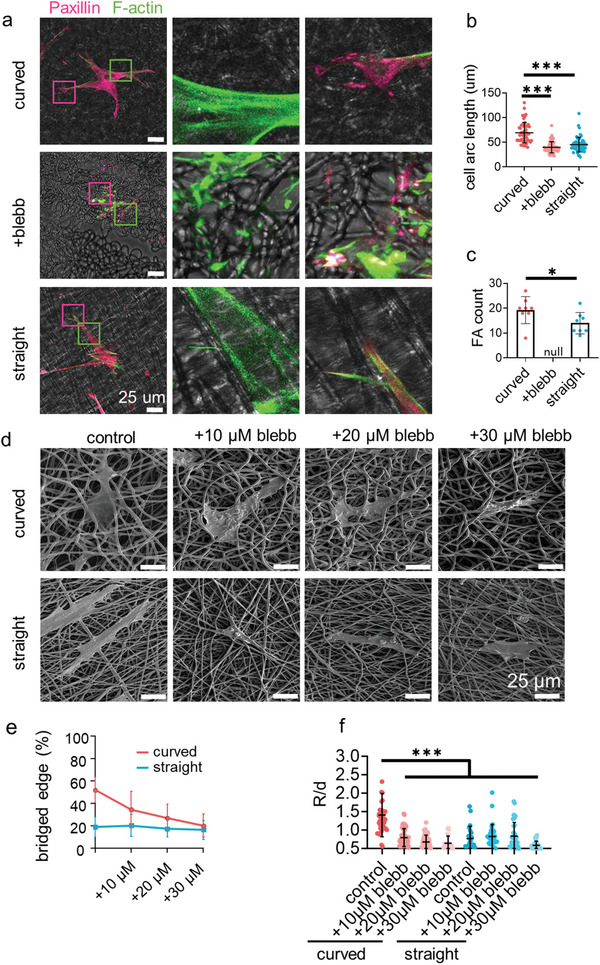
The stress fiber tension drives cells to bridge. a) The representative fluorescence images of F‐actin (green), paxillin(magenta) staining after 24 h of cell culture on the curved and straight fibers or on the curved fibers with blebbistatin (blebb, 20 µm) treatment. b) The length of the cell arc on the fibers (*n* = 50, two biological replicates). c)The number of focal adhesions in each single cell on the fibers (*n* = 10, two biological replicates). d) The representative SEM images and e) the percentage of the bridged edge (*n* = 15–30, two technical replicates) of the single cell treated with blebbistatin on the curved and straight fibers. f) The quantification of the *R*/*d* value of the cells treated with blebbistatin on the curved and straight fibers.

To explore the driving force for inducing cell bridge formation, we focus on the actomyosin cytoskeleton, which is the essential component of the molecular clutch to generate and transmit the traction force.^[^ ^14^ ^]^ As imaged by filamentous actin (F‐actin) staining, the actomyosin cytoskeleton is condensed along the arc of the cell bridges (**Figure** **5**a,b). As the actomyosin is the relatively stiff cytoskeleton,^[^ ^50^ ^]^ it applies large tension to resist the bend of the bridging cell edges. This observation explains the intracellular tension calculated by the 2D Laplace's law model.

The high actomyosin cytoskeleton tension may, in turn, drive cells to bridge on the curved fibers. To examine this hypothesis, we zoomed in the link between actomyosin stress fiber and the substrate fibers (Figure 5a,b; Figure S4, Supporting Information). Most of the stress fibers extended along the straight fibers. Meanwhile, some stress fibers near the cell edges extended to the tangent direction of the curved fibers, and most of the other stress fibers extended to the orthogonal direction of the curved fibers. The cell adhesive clusters on the substrate fibers were further investigated by paxillin immunostaining because the actomyosin stress fiber started to grow from the focal adhesion (FA) points.^[^ ^7^ ^]^ The focal adhesions (FAs) were observed to link the actomyosin stress fiber and the substrate fibers. On the straight fibers, the FAs extended along the fibers to form long clusters. On the curved fibers, the FAs could not extend with the bent fibers; and thus, were limited by the curvature of the fibers. However, the actomyosin tension helped to unfold talin to form more FA clusters on the curved fibers (Figure 5c), which was essential to activate the mechnotransduction signaling pathways.^[^ ^14^ ^]^ Therefore, the outline of cell bridge formation becomes clear. The FAs grew along the substrate straight fibers and guided the assembly of the actomyosin cytoskeleton to the fiber direction for aligning the cell body on the fibers. On the curved fibers, the FAs first grew on a short section of the fibers to initiate the assembly of the actomyosin cytoskeleton to the tangent direction of the fiber arc because the actomyosin cytoskeleton was rigid enough to resist the bending stress. It created the non‐adhesive region under cell bodies; and thus, the cells reshaped their bodies to cross different fibers forming cell bridges. Varying concentrations of blebbistatin (0, 10, 20, and 30 µm) were utilized to disassemble the actomyosin cytoskeleton to soften the cell edges. As displayed in the SEM images in Figure 5d; Figure S5, Supporting Information, the cell edges were gradually switched from forming bridges to attaching on the curved networks, while the contact of cell edges on the straight fibers was not affected. The percentage of the non‐adhesive edges finally decreased to the same level as on the straight fibers (Figure 5e). The *R*/*d* ratio of the non‐adhesive bridge on the curved fibers also decreased dramatically after blebbistatin treatment (Figure 5f). This result confirmed the role of actomyosin cytoskeleton on inducing cell bridge formation.

### 2.6 Cells Form Bridges in the Connective Tissue of Periodontal Ligament

**Figure 6:**
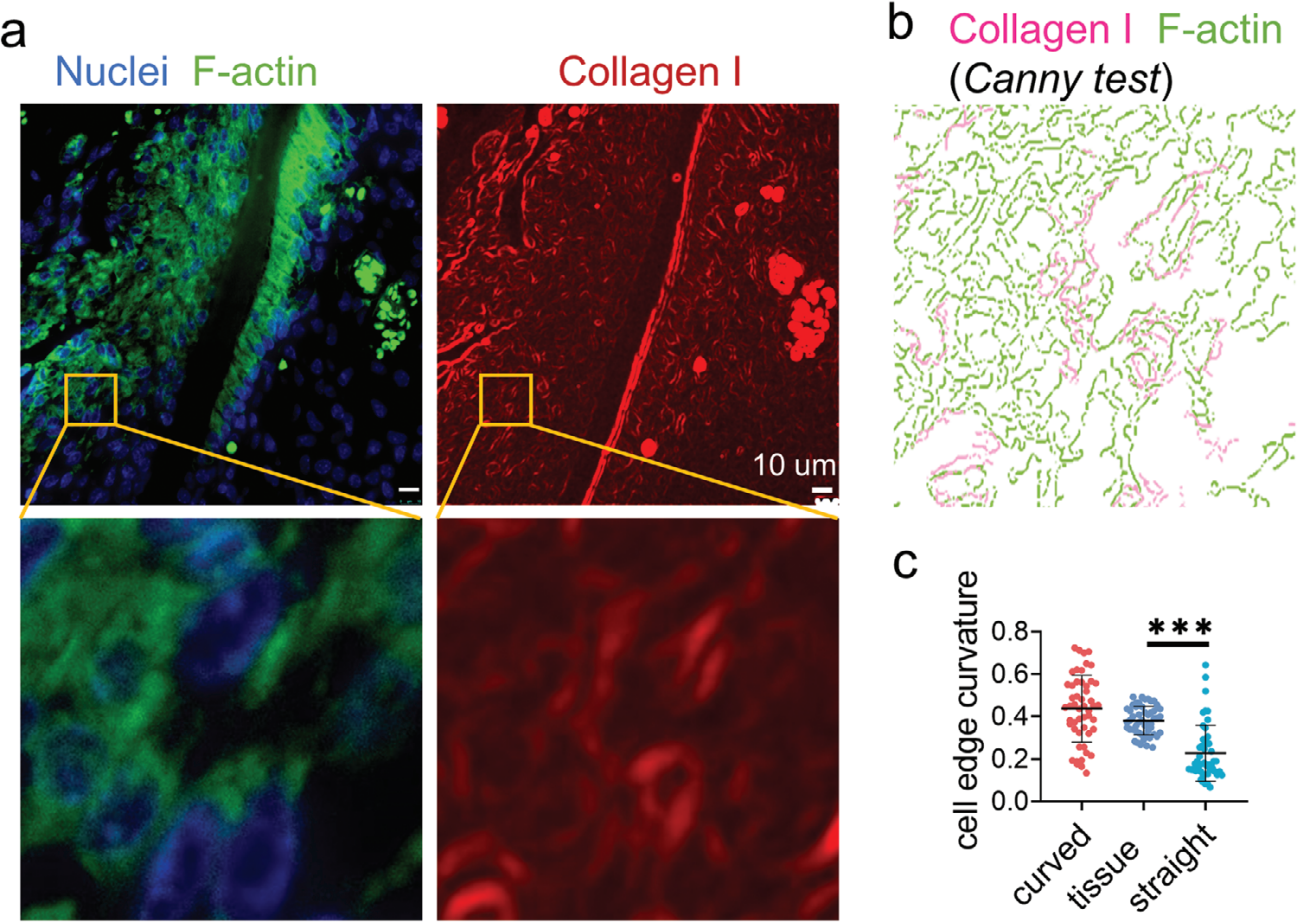
Immunofluorescence staining displays widely distributed cell bridges in the periodontal ligament. a) The representative fluorescence images of nuclei (blue), F‐actin (green), and collagen I (red) staining of the mouse periodontal ligament. b) Canny edge test image of the yellow box area in (a). The magenta and green represent the collagen I and F‐actin, respectively. c) The average curvature of the cell edges (*n* = 50, two technical replicates) of the cells in periodontal ligament and cultured on the artificial fibers.

The cell bridge phenomenon was also observed in the connective tissue of mouse periodontal ligament. The Canny edge detector was used to plot the cell edges in the fluorescent images.^[^ ^51^ ^]^ It can be observed that the cells crossed several curved structures (**Figure** **6**a,b; Figure S6, Supporting Information). The curvature of the cell edges in periodontal tissues showed excellent consistency with the cells on the curved fibers (Figure 6c). This suggests the curved morphology in vivo is a mechanical regulator for cell behavior control as the cells in connective tissue normally require intracellular force.

## 3 Discussion

**Figure 7:**
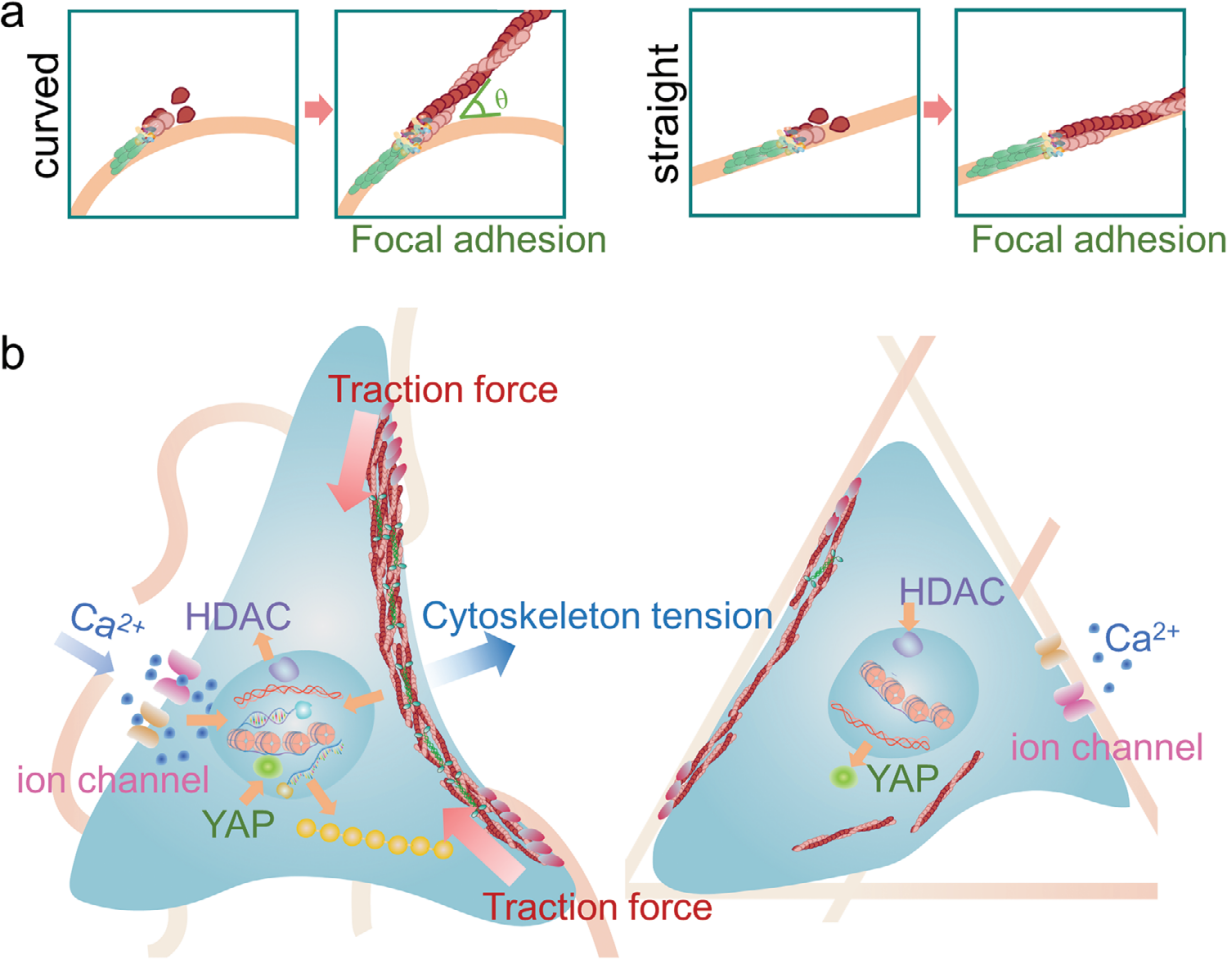
Schematic illustration of the cell adhesion characteristics on the curved and straight nanofiber networks. a) The polymerization of the actomyosin fibers on the curved and straight fibers. b) On the curved (left) fibers, the fiber curvature induces the formation of the non‐adhesive bridges, which promotes PDLSC mechanotransduction through the condensed actomyosin stress fibers.

The extracellular matrix (ECM) is composed of fibrous proteins that are assembled to build the tissues leading to the diversification of the tissue structures. The architecture of the fibrous protein is not always continuous, which forms randomly distributed micron‐sized gaps.^[^ ^52^, ^53^, ^54^ ^]^ It is clear that the ECM fibrous structure is crucial for the guidance and maintenance of the cellular functions. In this study, we produced curved nanofibers to mimic the structure of the natural ECM in periodontal ligament. The curved fibers promoted cell proliferation and osteogenic differentiation (**Figure** **7**). The detailed study about the cell mechanosensing revealed that the cell boundary always crossed several curved fibers as the bridge but was mostly parallel with the surrounding straight fibers. The cells on the curved fibers had high percentage of unattached boundaries forming free arcs and bowing inward with large radius. According to the Laplace's law model, this morphology indicated greater intracellular tension, which was generated from the stiff actomyosin stress fiber near the cell edges. Previous study has proven that the long actomyosin cytoskeleton concentrated at the reign with high mechanical tension, while it disaggregated with low mechanical tension.^[^ ^55^ ^]^ The high tension initiated the mechanotransduction pathways as well as opened the ion channels on the cell membrane. The chromatin was thus remodeled via nuclear mechanotransduction to activate the transcription of the proliferation and osteogenic differentiation related genes with the association of calcium ions.

The initially formed FA clusters adhered and grew along the fibers, which led the actomyosin cytoskeleton to extend in the same direction of FA growth.^[^ ^56^, ^57^ ^]^ The stiff actomyosin bundles could not be bent to match the curved fibers, so the cell bodies could not permanently attach to the fibers and had to bridge between the fibers (Figure 7a). The cell edges bowed inward due to the lack of adhesive support but were resisted by the concentrated actomyosin stress fibers. The reshaped stress fibers further unfolded adhesive structural proteins and stabilized the FAs on the curved fibers (Figure 7). Thus, the cell activated higher levels of intracellular traction force, achieving a suspended bridge state. In contrast, the FA‐guided actomyosin bundles extended in the fiber direction without deflection on the straight nanofibers; so, the cell edges could easily attach to the fibers.

In the bridge structure, the actomyosin cytoskeleton can migrate from the adjacent adhesive fibers to the middle of the non‐adhesive bridge.^[^ ^58^ ^]^ This dynamic process makes an equilibrium of tensional forces within a cell. The traction force and tension in actomyosin are exerted by myosin II motor per se. Therefore, the unattached bridges stimulate the contraction of myosin II between the two adhesion points. Kilian et al. also put forward the conclusion that cells had stronger osteogenic differentiation in the region with the non‐adhesive patterns.^[^ ^59^ ^]^ Schwarz et al. proposed that the distribution and size of cell adhesion were related to the continuity of the adhesion pattern. The cell force was concentrated at the vertices of the cell body and contracted along the cell profile.^[^ ^60^ ^]^ Sheetz et al. observed the actomyosin‐based sarcomere‐like contractile units crossed the discontinuous adhesive regions.^[^ ^61^ ^]^ In fact, many cell types have been reported to form bridge patterns connecting the discontinuous adhesive interface in either 2D or 3D matrixes.^[^ ^62^, ^63^, ^64^, ^65^, ^66^ ^]^ Therefore, cell bridge formation may be a common phenomenon in natural tissue to regulate cell mechanotransduction.

The actomyosin contraction is transmitted to the cell membrane to modulate the activity of ion channels including Piezo1 and TRPV4 to control the Ca^2+^ influx.^[^ ^67^, ^68^ ^]^ The ion channel Piezo1 perceives membrane tension and curvature. Its orientation domain senses cell membrane curvature for switching its conformation to the active state.^[^ ^67^ ^]^ As proven above, the high cell edge curvature and tension assisted Piezo1 activation for acquiring calcium ions for cells. The actomyosin traction force and tension can transmit to cell membrane on the one hand, and to the cell nucleus on the other.^[^ ^14^ ^]^ The nucleoskeleton Lamin A/C senses the actomyosin contraction and converts the mechanical stimuli to regulate the chromatin accessibility and transcriptional activity.^[^ ^17^ ^]^ The calcium ion is involved in the process of force transmission and transduction. The calcium ion homeostasis mediates the energy production in mitochondria to support cellular contraction.^[^ ^69^ ^]^ The calcium ion influx modulates the activities of the Rho GTPase pathway, which affects the local assembly of F‐actin and stabilizes the link between myosin II and actin filaments.^[^ ^70^, ^71^ ^]^. In addition, the intracellular force determines the karyoplasmic ratio of calcium ions, and the nuclear calcium ions regulate gene expression via activating calmodulin‐dependent kinase and phosphorylating transcription factors.^[^ ^72^ ^]^ Therefore, the calcium ion stimulation and nuclear mechanics synergize to drive the cell force downstream cell functions.

In addition, although the electrospinning technology has been widely used to fabricate ECM‐mimicking biomaterials, there are few reports on the technology of producing curved nanofibers. Some studies that performed low‐temperature electrospinning only focus on matrix porosity instead of fiber topology.^[^ ^73^ ^]^ We obtained the curved nanofibers just by adjusting the temperature and receiving speed. It offers a new tool to mimic the ECM structures.

## 4 Conclusion

In summary, we have developed a simple low‐temperature and low‐speed electrospinning technology to prepare curved nanofiber structures that mimic a class of extracellular matrix. The curved nanofiber induces the cell to form discrete adhesion, and the straight network induces cells to form continuous adhesion along with the fiber structure. This curved nanofiber structure significantly stimulates cellular mechanotransduction through cell bridge formation, and therefore, promotes the osteogenic differentiation and proliferation activity of the cells. The non‐adhesive bridges activate cell actomyosin aggregation and contraction, which initiate the mechanosensing and mechanotransduction signaling pathway: discrete adhesion sites‐actomyosin cytoskeleton assembly and aggregation‐Lamin A/C assembly‐chromatin accessibility. This curved matrix could be used to supplement the database of the ECM‐mimicking biomaterials and enrich the knowledge of cell mechanosensing and tissue development.

## 5 Experimental Section

### Masson's Trichrome Staining

Postnatal 4 to 5 weeks CD‐1 male mice were euthanatized by CO_2_, followed by cervical dislocation. The study was approved by the Ethical Committee of West China Hospital of Stomatology, Sichuan University (WCHSIRB‐D‐2018‐093). The mandibles were collected and fixed with 4% paraformaldehyde. The fixed tissues were immersed in 0.5 m EDTA (pH 7.4) for 2 weeks for decalcification and were dehydrated with a graded alcohol series and embedded in paraffin. Paraffin sections (5 µm) were used for Masson's trichrome and immunohistochemical staining.

The Masson's trichrome staining was conducted as the protocol of Masson's trichrome staining Kit (G1006, Servicebio). After dewaxing and rehydration, the sections were incubated with solution A overnight (15 h) and then were heated at 37 °C for 30 min. The sections were immersed in the mixed preheated solutions D and F for 1 min, and then were washed with running water. After infiltrating with solution D for 6 min and solution E for 1 min, the sections were slightly treated with excess solution E for 2–10 s. Finally, the sections were dehydrated and covered with natural balsam for microscope observation.

### Scanning Electron Microscopy (SEM)

The sections were heated in an oven at 60 °C for 45 min and were dewaxed with xylene for 10 min twice. Then, the sections were dehydrated in 80%, 90%, and 100% alcohol for 3 min, respectively, and mounted on holders and coated with gold/palladium. Finally, the samples were examined with scanning electron microscope.

### Preparation of Curved and Straight Nanofiber Matrix

A 10% electrospinning solution was prepared by dissolving polycaprolactone (PCL) (440744‐500G, Sigma) in 10 mL of 1,1,1,3,3,3‐Hexafluoro‐2‐propanol (H107501‐500 g, Aladdin) at 37 °C with constant stirring for 8 h. The prepared polymer solution was electrospun by using a conventional electrospinning device with the following parameters: 17 kV of static electric voltage, 25 cm of air gap distance, and 0.01 mL min^−1^ flow rate of solution. For straight nanofiber membrane, the rotating receiver speed and temperature were 1500 rpm and 37 °C. For curved nanofiber membrane, the prepared polymer solution was precooled at −20 °C; the rotating receiver speed and temperature were 400 rpm and 0 °C. After 5 h of electrospinning, the formed nanofiber membrane was gathered.

Before seeding cells on the surface of the matrix, the fibers were sterilized with 75% ethanol for 30 min followed by thorough PBS washing.

### Material Characterizations

The surface morphology was characterized by field emission scanning electron microscope (Nova NanoSEM450, FEI, USA). Before observation, all samples were plated with platinum. The Young's modulus of the sample was characterized by bio‐nanoindenter (Piuma Chiaro, Optics 11, Netherlands). The specific surface area of the sample was determined by adsorbing bovine serum albumin (BSA) on the surface of the sample. These samples were treated with fluorescein‐labeled BSA (1 mg mL^−1^, BSA‐fitc, SF063, Solarbio, China) for 1 h. The BSA could only form a single layer on the surface of the sample. The specific surface area was calculated by the fluorescence intensity of the surface.

### Cell Culture and Inhibitor Treatment

HPDLSCs were isolated, as previously described,^[^ ^74^ ^]^ which was approved by the Ethical Committee of West China Hospital of Stomatology, Sichuan University (WCHSIRB‐D‐2021‐051). The healthy extracted premolars from the orthodontic patients were selected and saved with 10% penicillin–streptomycin solution. The middle 1/3 tissue of the tooth root was scraped into DMEM (Gibco, C11995500BT) containing 1% penicillin–streptomycin. The tissue was centrifuged at the speed of 1000 r min^−1^ for 5 min. Following this, the tissue was digested with the mixed solution of type I collagenase (3 mg mL^−1^, Gibco, 17100017), and type II Disperse enzyme (4 mg mL^−1^, Gibco,17105041) in water shaker bath at 37 °C for 45 min. After the digestion and centrifugation, the periodontal ligament tissue was cultured in T25 culture flasks with 20% FBS in DMEM medium. The obtained cells were cultured under 37 °C, 5% CO_2_, and humidified conditions. The 3rd generation of the cells was used for experiments. To explore the multi‐differentiation potential, the cells were cultured in the osteogenic differentiation medium, chondrogenic differentiation medium, and adipogenic differentiation (Cyagen, China) for corresponding time. The results of ALP staining, Alcian blue staining (Cyagen OriCell, ALCB‐10001), and Oil Red (Cyagen OriCell, OILR‐10001) staining showed that PDLSCs have the multipotential differentiation with the mesenchymal stem cell characteristics (Figure S7, Supporting Information).

In the inhibitor experiments, the cells were placed on curved or straight nanofiber networks at a density of 5000 cells cm^−2^, and the (‐)‐Blebbistatin (20 µm, MERCK, B0560) was added during the whole cell culture period.

### Immunofluorescence Staining and Microscopy

The samples were washed twice with PBS and then fixed with 4% paraformaldehyde at room temperature for 30 min. The samples were then washed with cold PBS three times. Cells were permeated with 0.25% v/v Triton‐X 100 PBS for 10 min at room temperature and then washed with PBS three times. Non‐specific antibody binding was blocked by incubating samples with 1% w/v bovine serum albumin (BSA) in PBST (0.1% v/v triton‐x 100 in PBS [PBST]) for 60 min at room temperature. Next, the samples were washed briefly with PBST and incubated overnight with primary antibody at 4 °C. After the above process, the samples were washed twice with PBST and three times with PBS. The samples were then incubated with secondary antibody, DAPI (vectorlabs, H‐1200), and fluorophore‐labeled phalloidin (Thermo Fisher Scientific, A12379) at room temperature for 60 min, then washed twice with PBST and three times with PBS. Immunofluorescence images were obtained by Leica DMi8 microscope and Leica St5 laser scanning confocal microscope The spread area, average orientation angle, and other factors were measured by Fiji software, and each sample contained at least 50 cells.

The primary antibodies and corresponding concentrations used were rabbit monoclonal anti‐myosin IIa (Cell signaling, 14611 s, 1:200 dilution), mouse monoclonal anti‐Paxillin (BD Transduction Laboratories, 612405, 1:200 dilution), rabbit monoclonal anti‐HDAC1 (D5C6U) (Cell signaling, 34589S, 1:200 dilution), rabbit monoclonal anti‐TRPV4 (Abcam, ab39260, 1:200 dilution), rabbit monoclonal anti‐Piezo1 (Alpmone labs, APC‐087, 1:200 dilution), rabbit monoclonal anti‐RNA polymerase II CTD repeat YSPTSPS (phospho S2) (Abcam, ab193468, 1:200 dilution), rabbit anti‐YAP (Cell signaling, 4912S, 1:200 dilution), and mouse monoclonal anti‐Lamin A/C (Novus biologicial, NB100‐74451, 1:200 dilution). Secondary antibodies used were anti‐Rabbit Alexa Fluor 568 (Thermo Scientific, A‐11011, 1:500 dilution) and Goat anti‐Mouse Alexa Fluor 647 (Thermo Scientific, A‐32728, 1:500 dilution). The living cell membrane dye DiO (DiOC18(3)) (US EVERBRIGHT, D4007) was used to image the cells for traction force microscopy.

### Western Analysis

After 24 h of culturing, fibrous matrices were washed with cold PBS two times. Cells were lysed into RIPA Lysis Buffer (RIPA Lysis Buffer [Strong], K1020, APExBIO) with Minitab Protease Inhibitors (K1007, K1015A, K1015B, APExBIO).

The protein content of lysates was determined with the bicinchoninic acid (BCA) protein assay (P0010S, Beyotime Biotechnology). 25 µg of protein was separated by SDS‐PAGE electrophoresis on 4–12% Tris‐Glycine Gels (SurePAGE, Bis–Tris, 10 × 8, 4–12%, ten wells, M00652 GenScript Biotech Corporation), and transferred to PVDF membranes (IPCH00010, Merck). Membranes were blocked (P0239, Beyotime Biotechnology) and then incubated with primary antibodies overnight at 4 °C, washed in TBST twice, and then incubated with HRP‐tagged secondary antibodies at room temperature for 60 min, washed in TBST twice, and followed by UltraSignal Hypersensitive ECL chemiluminescence substrate (4AW011‐100, 4A Biotech). Blots were developed using Fluorescence and chemiluminescence imaging systems (ChemiScppe 6100, Clinx). GAPDH was used as an internal loading control. Digital images of Western Blots were quantified using Fiji software.

The primary antibodies and corresponding concentrations used were rabbit monoclonal anti‐myosin IIa (Cell signaling, 14611s, 1:200 dilution), rabbit monoclonal anti‐HDAC1 (D5C6U) (Cell signaling, 34589S, 1:200 dilution), rabbit monoclonal anti‐Piezo1 (Alpmone labs, APC‐087, 1:200 dilution), mouse monoclonal anti‐Lamin A/C (Novus biologicial, NB100‐74451, 1:200 dilution), and rabbit polyclonal anti‐GAPDH (Signalway Antibody LLC, 21612, 1:5000 dilution). Secondary antibodies used were Goat Anti‐Mouse IgG Secondary Antibody HRP Conjugated (Signalway Antibody LLC, L3032, 1:10 000 dilution) and Goat Anti‐Rabbit IgG Secondary Antibody HRP Conjugated (Signalway Antibody LLC, L3012, 1:10 000 dilution).

### Traction Force Microscope

Nanofiber membranes were prepared by mixing 0.5 µm fluorescent carboxylated polystyrene beads (Latex beads, carboxylate‐modified polystyrene, fluorescent red, Sigma, L3280‐1ML) in PCL electrospinning polymer solution. The fluorescence microscopy was used to take images of the nanobeads and the adhered cells. Last, cells were removed by treating with 5% SDS on the microscope table for 10 min. These images were used to define the original location of the fluorophore‐labeled nanobead. The Fiji plugin “Align Slices in stack” was used to correct the experimental drift of the sample. Subsequently, Fiji's Particle Image Velocimetry plug‐in was used to calculate the displacement field in the cell region.

### Osteogenic Differentiation on Nanofiber Matrix

PDLSCs were seeded at a density of 5000 cell cm^−2^ on the curved and straight fibers in human dental pulp stem cell osteogenic differentiation medium (Coage, HUXDP‐90021, China), which contained 10% fetal bovine, 1% penicillin–streptomycin, 2 mL *β*‐glycerophosphate, 2 mL glutamine, 400 µL ascorbate, and 20 uL dexamethasone. The induction medium was only used in the differentiation experiments in Figure 2. The cells were cultured for 7 and 14 days, and the osteogenesis was detected by alkaline phosphatase (ALP, Beyotime, C3206) staining and Alizarin red S (ARS, Cyagen, ALIR‐10001) Staining. The cells for 14 and 21 days were fixed with 4% formaldehyde (Biosharp, BL539A) for 30 min and stained with 0.1% ARS (at room temperature for 1 h, and then the sample was washed with PBS and to visualize the mineral deposition. For ALP staining, the fixed cells were stained with BCIP/NBT Alkaline Phosphatase Color Development Kit (Beyotime, Biotechnology) according to the manufacturer's instructions for 30 min and washed with PBS.

### Quantitative Real‐Time Polymerase Chain Reaction Analysis (qRT‐PCR)

The RNAs were isolated by TRIzol reagent (Thermo, 15596026) and a PrimeScript RT reagent kit (Takara, Code No. RR820A) was used for DNA synthesis. The qRT‐PCR was conducted with the QuantStudio 3 Real‐Time PCR Systems (Thermo Fisher Scientific) to detect gene expression. The primers were synthesized by Sangon Biotech (Shanghai). TB Green Premix Ex Taq II (Takara, Japan) was used for reaction. Gene expression level was normalized to glyceraldehyde 3‐phosphate dehydrogenase (GAPDH). The relative expression was calculated by the formula 2^−ΔΔ^Ct. The primer sets used are in Table S1, Supporting Information.

### Chromatin Condensation Parameter (CCP)

To generate chromatin condensation parameters (CCP), the gradient‐based Sobel edge detection algorithm was performed to measure the edge density of individual nuclei MATLAB in MATLAB R2016a.^[^ ^42^ ^]^

### Decellularization

Extracted premolars of orthodontic patients were collected for preparing the samples for decellularization, which was approved by the Ethical Committee of West China Hospital of Stomatology, Sichuan University (WCHSIRB‐D‐2021‐051). Teeth were washed with phosphate‐buffered saline (PBS), and then, the teeth were cut with DTX‐5 (Veiyee, China) to obtain slices of ≈1 mm thickness without pulp tissue. Those tooth slices were frozen at −80 °C for use. The tooth slices were defrosted at room temperature and then submerged in 0.5% chloramine‐T (Sigma–Aldrich, St. US) for 2 h at 4 °C and washed with PBS. Tooth slices were incubated in 1% Triton X‐100 for 24 h, and then, 1% sodium dodecyl sulfate (SDS, Sigma–Aldrich, St. US) for 24 h to decellularize the samples. This cycle was repeated three times at room temperature with constant gentle agitation of the samples in a shaker (Haimen Kylin‐Bell, China). At the end of each cycle, samples were rinsed with 10% ethylenediaminetetraacetic acid (EDTA, ThermoFisher Scientific Co., Houston, TX, USA) having a level of ≈PH8 for 5 min, and then, were rinsed three times with PBS for 10 min each time. Last, the decellularized PDL was obtained. The decellularized tooth slices were dehydrated with gradient ethanol dehydration before scanning electron microscopy.

### Images and Model Analysis

The redefined Laplace model was utilized to calculate the cellular tension in the bridge region. A custom‐written code in MATLAB R2016a was performed to depict the cell edge on related substrates to calculate the cell edge based on the collagen I and F‐actin immunostaining of the tissue samples (Programs S1–S4, Supporting Information).

### Statistics

Statistic analysis was performed by using GraphPad Prism 8. One‐way analysis of variance (ANOVA) and posthoc Tukey's multiple comparison test were performed on the experiment date. *p* < 0.05 (*p < 0.05, **p < 0.01, ***p < 0.001, and ****p < 0.0001) demonstrated the significance. All statistical conclusion was expressed as mean ± standard deviation. Min–max normalization was used to normalize scores into [0, 1]; the maximum value in a set of data was defined as 1, and the minimum value was defined as 0.

## Conflict of Interest

The authors declare no conflict of interest.

## Supporting information

Supporting Information

## Data Availability

The data that support the findings of this study are available from the corresponding author upon reasonable request.
