# Supplementary material for: Curved Nanofiber Network Induces Cellular Bridge Formation to Promote Stem Cell Mechanotransduction
Source: Adv Sci (Weinh). 2022 Nov 16;10(3):2204479. doi: 10.1002/advs.202204479 (PMC9875655; doi:10.1002/advs.202204479)
Supplement: Supplementary file 1 — Supporting Information [file ADVS-10-2204479-s001.pdf]

ADVANCED SCIENCE
Open Access

Supporting Information

for *Adv. Sci.*, DOI 10.1002/advs.202204479

Curved Nanofiber Network Induces Cellular Bridge Formation to Promote Stem Cell Mechanotransduction

Qian Sun, Fang Pei, Man Zhang, Bo Zhang, Ying Jin, Zhihe Zhao and Qiang Wei**

Supporting Information

Curved Nanofiber Network Induces Cellular Bridge Formation to Promote Stem Cell Mechanotransduction

Qian Sun,[a, b, +] Fang Pei,[a, b, +] Man Zhang,[c] Bo Zhang,[a] Ying Jin,[a, *] Zhihe Zhao,[a] Qiang Wei[b, c,*]

a. Department of Orthodontics, State Key Laboratory of Oral Diseases, National Clinical Research Center for Oral Diseases, West China Hospital of Stomatology, Sichuan University, Chengdu 610041, China.

b. College of Polymer Science and Engineering, State Key Laboratory of Polymer Materials and Engineering, Sichuan University, Chengdu 610065, China.

c. College of Biomedical Engineering, Sichuan University, Chengdu, 610065, China

Email: wei@scu.edu.cn (Q. W.), jinying@scu.edu.cn (Y. J.)

+ Q. S. and F. P. contributed equally to this work.

Specific surface area of the curved and straight fibers

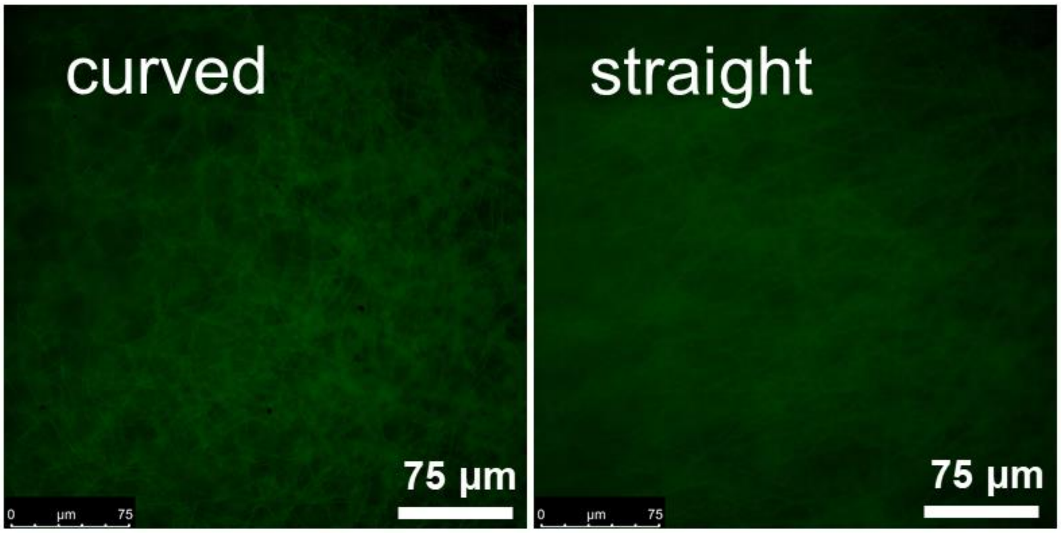

Figure S1. The fluorescent images of the FITC-BSA adsorbed on the curved and straight fibers.

The cell spreading of PDLSCs on the curved and straight fibers.

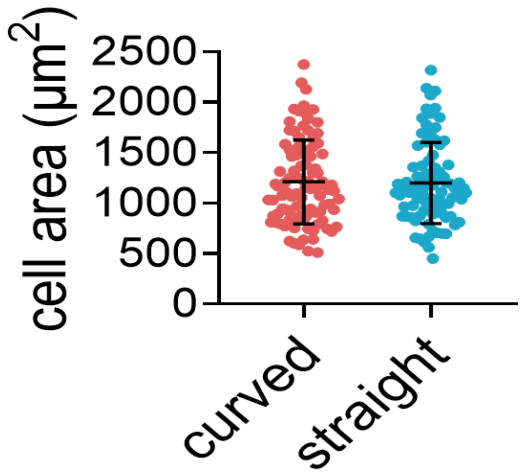

Figure S2. Cell spread area of the cell cultured on the related surfaces for 24 h (n = 100, 2 technical replicates).

Cells bridge on the curved fibers.

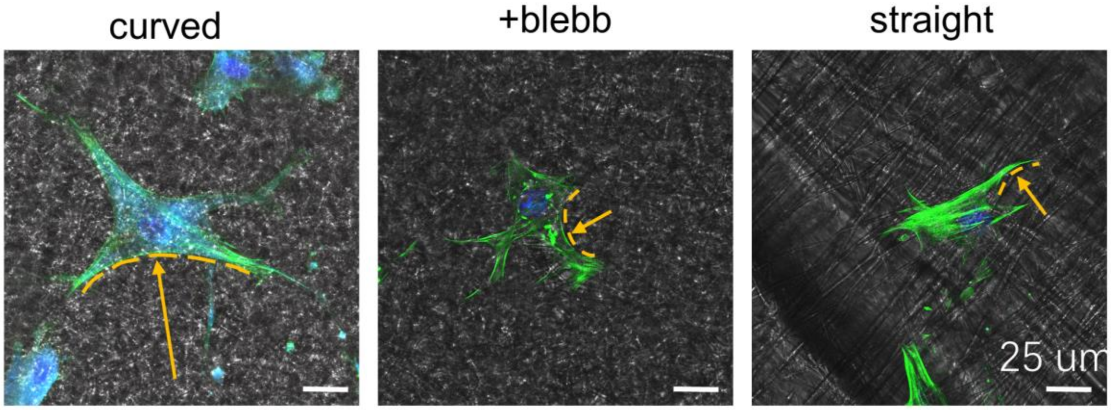

Figure S3. The representative images of PDLSCs on the curved and straight fibers. Green represents F-actin, blue represents nuclei. The dotted line defines the cell bridge and the yellow arrow indicates the radius of curvature.

The stress fiber tension drives cells to bridge.

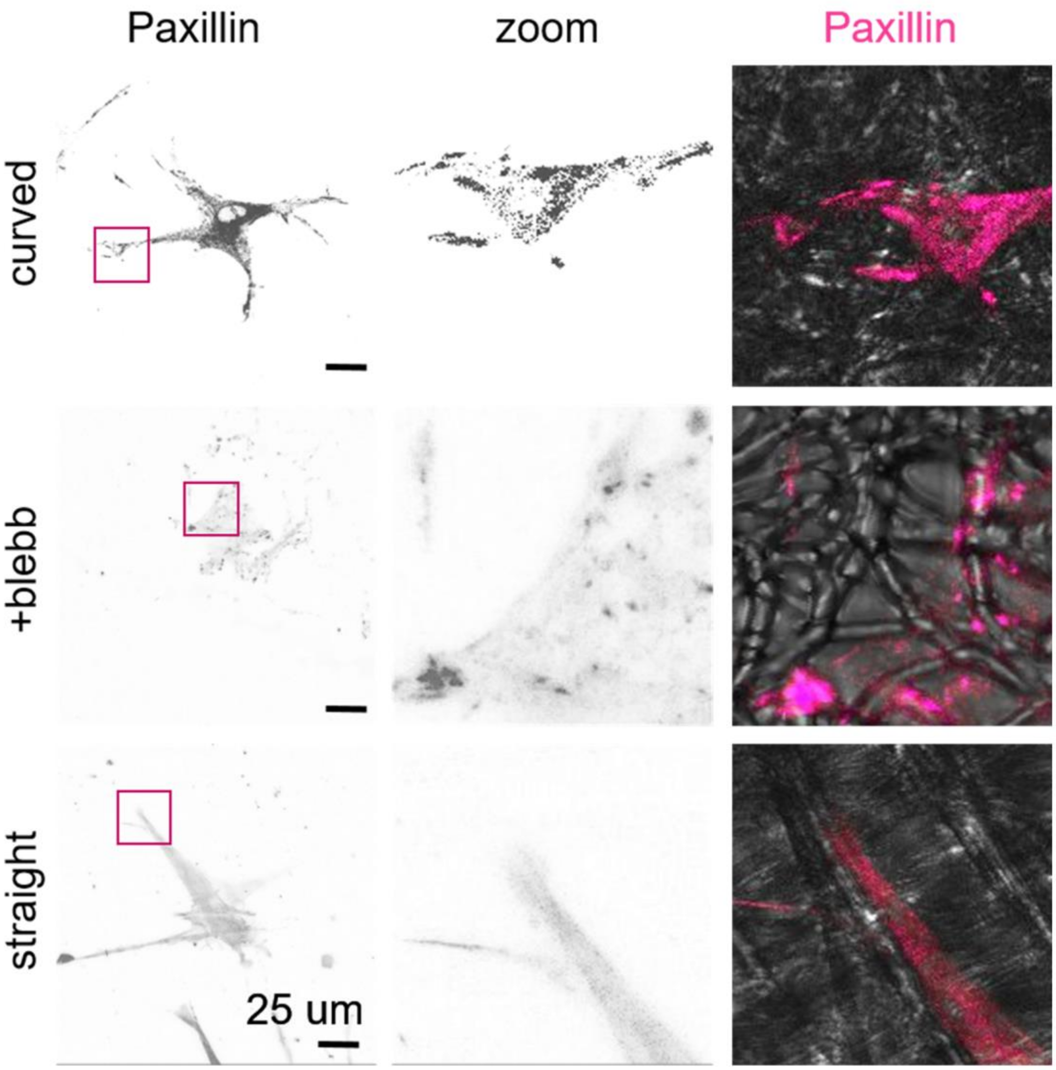

Figure S4. The representative fluorescence images of paxillin staining after 24 h of cell culture on the curved and straight fibers or on the curved fibers with blebbistatin (blebb, 20 μM) treatment.

Re-marked the edge of the cell adhesive and non-adhesive regions.

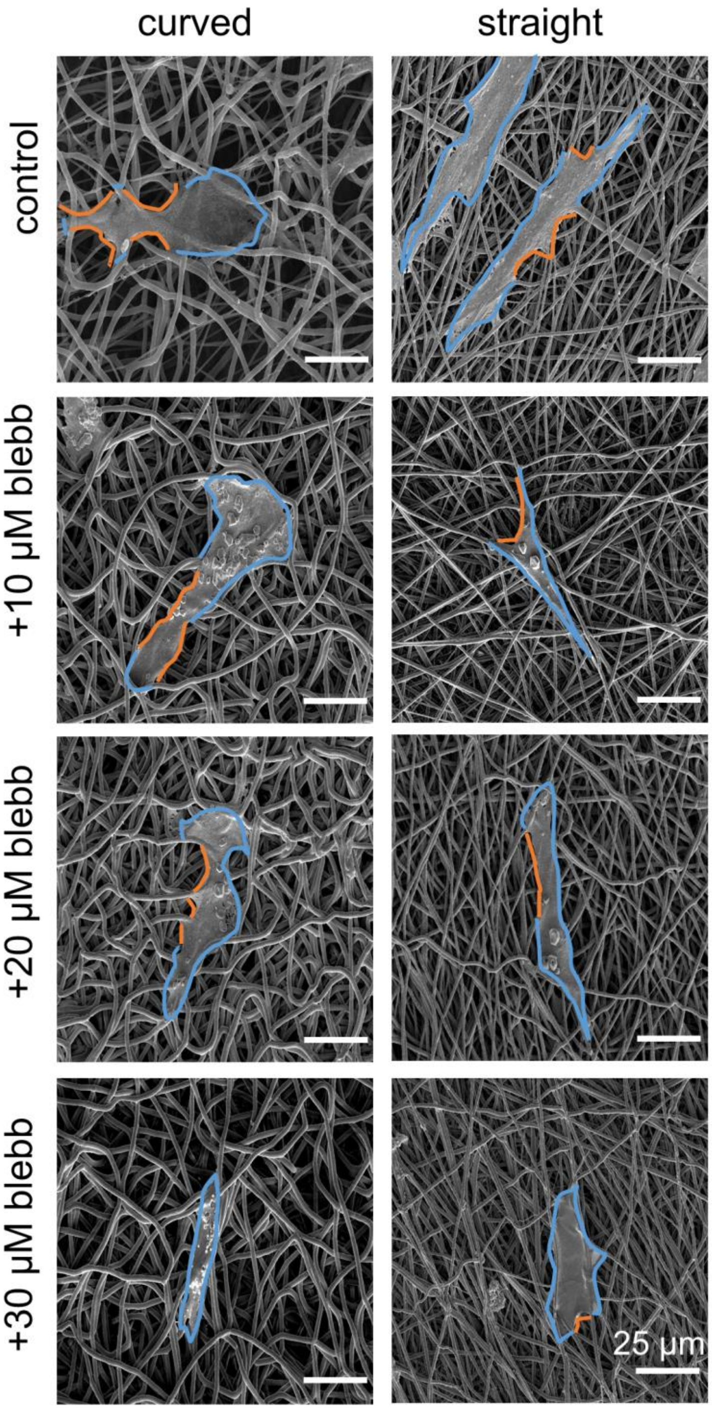

Figure S5. The representative SEM images of the single cell treated with blebbistatin on the curved and straight fibers, as indicating the adhesive edge in blue and non-adhesive edge in orange.

Cell bridges in the periodontal ligament.

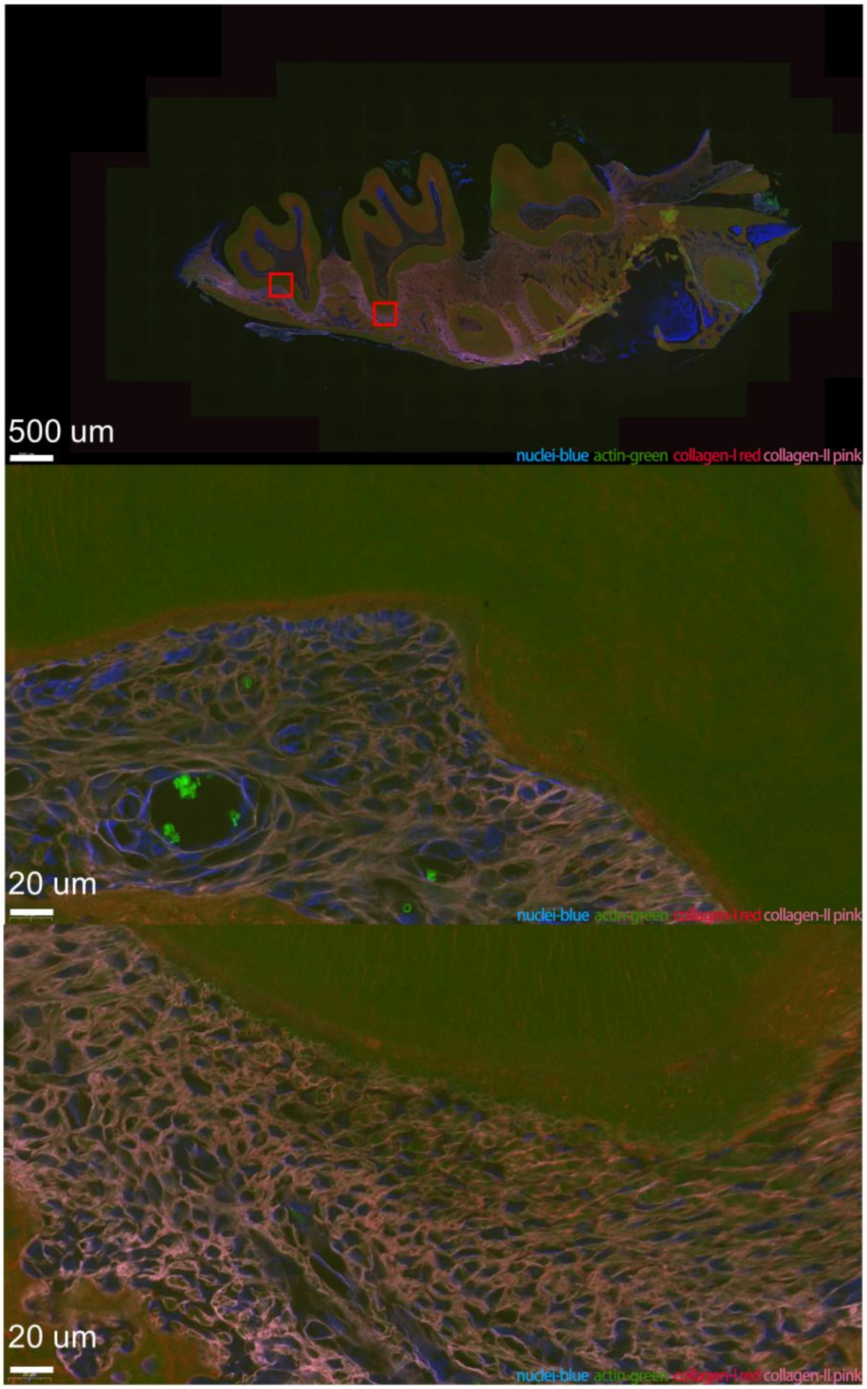

Figure S6. The representative fluorescence images of nuclei (blue), F-actin (green), collagen I (red) and collagen II (pink) staining of the rat periodontal ligament.

The differentiation potencies of the derived PDLSCs.

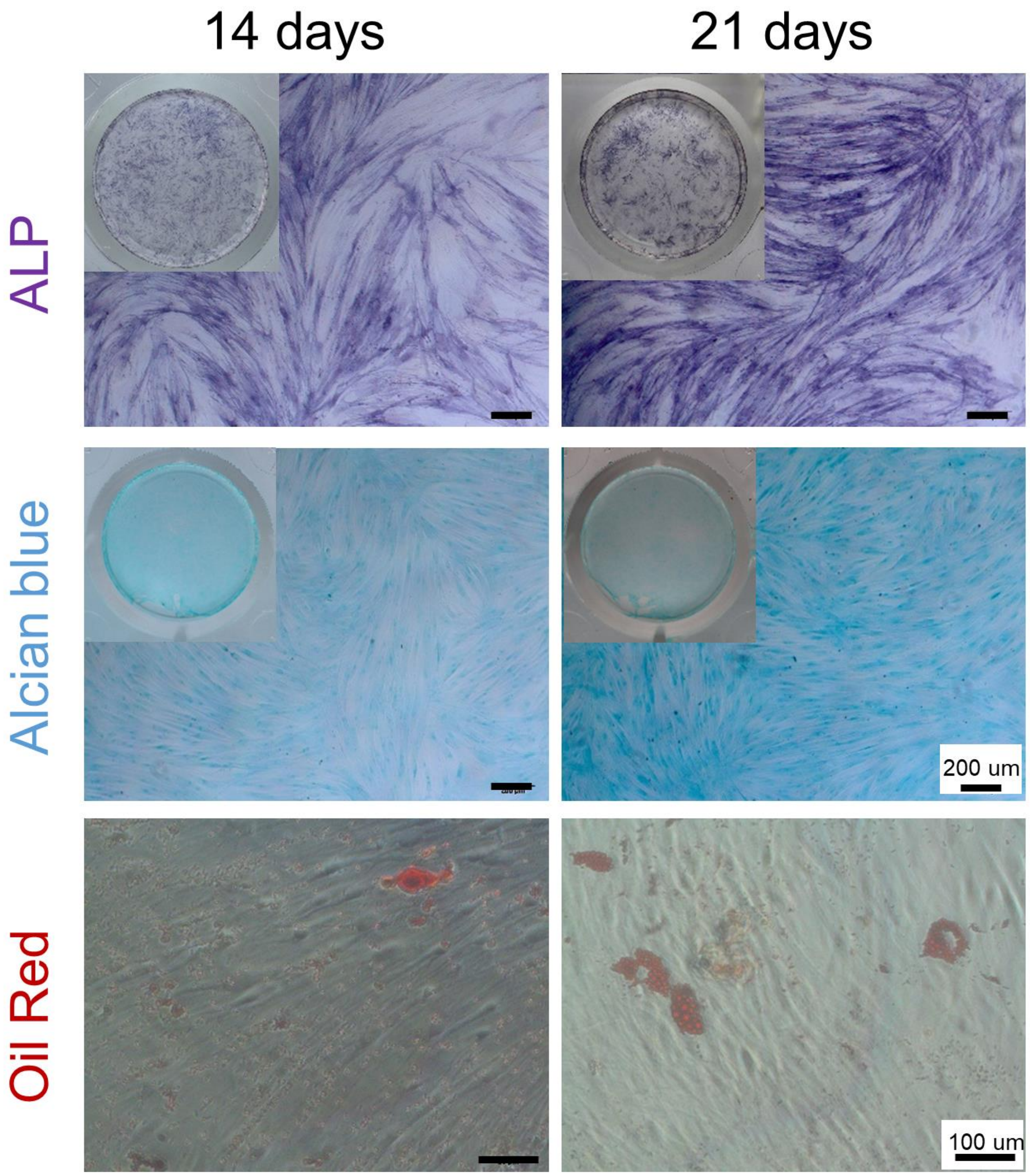

Figure S7. Cells derived from the healthy extracted premolars tissue are well differentiated towards osteogenesis, chondrogenesis, and adipogenesis.

Uncropped Western blots.

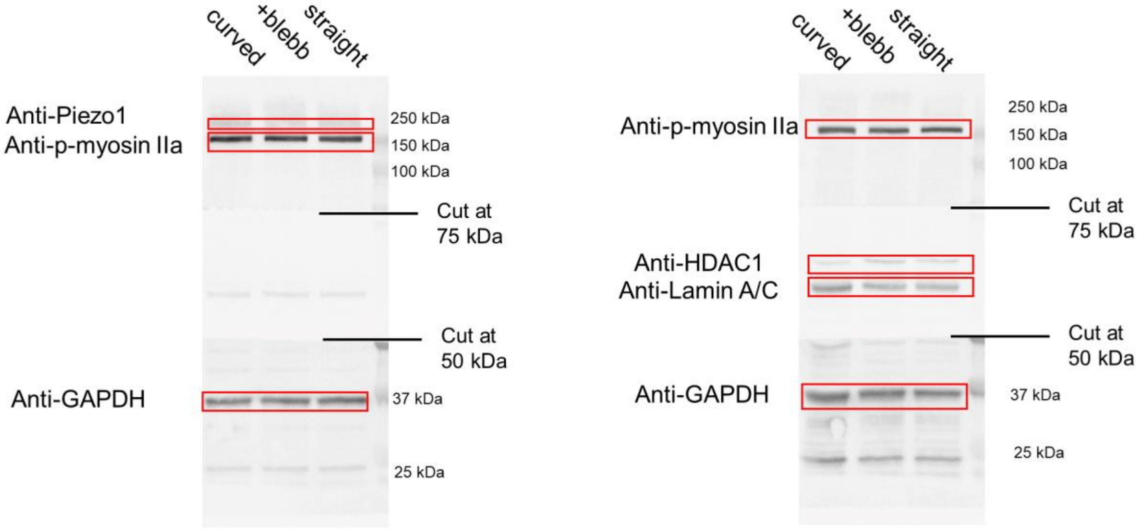

Figure S8. Full scan of uncropped membrane in the main figure. The membranes were cut at the indicated position before staining.

MATLAB Program 1. Representation of cell edge and curvature:

The main program:

```
clear
clc
x = xlsread('X-Coordinate (um)') % Each x coordinate of the scan point%
y = xlsread('Y-Coordinate (um)') % Each y coordinate of the scan point%
z = xlsread('Point Curvature (um-1)') % Each curvature coordinate of the scan point%

a = mean(x)
b = mean(y)
plot3(x, y, z) % Draw 3D images%

axis equal

hold on
scatter(a,b)% Mark the center of the cell%
hold on
patch(x,y,z,z, 'edgecolor','flat', 'facecolor','none')% Give a gradient color to the curvature%
view(3); grid on; colorbar

grid off
```

MATLAB Program 2. Representation of cell edge vertex:

The main program:

```
clear
clc
z = xlsread ('Point Curvature (um-1)') % Each curvature coordinate of the scan point%
hold on
```

MATLAB Program 3. Laplace model:

The main program:

```
clear
clc
 k = 5;%one actomyosin motor force,free parameter%
i = 1;%free.parameter,L0,fraction%
x = 2:0.1:75; %range of distance between one cell bridge,l%
n = numel(x)-1;
 y = 2:98/n:100; %range of curvature radius,R%
 y1 = 1./y;%count backwards %
 y2 = transpose(y1);%transposition %

 %myosin density caculation%
  m1 = 5;%myosin density free parameter 1%
 m2 =1.2;%myosin density free parameter 2%
 m3  = m1 * y.^m2;%myosin density, is a function of myosin density%
 m4  = transpose(m3);
 m   = repmat( m4 ,1,n+1 );

% Calculation process : R-d-F%%
 a1 = y2 * x;%l/R%
 angle1 = 2 * asin(a1./2);
 y3 = diag(y);%Create diagonal matrix%
 c  = y3 * angle1;
 d  = i./c;
 F  = 1./(1 + d);% ignore myosin density change,R-d-F%
 F1 = F .* m;% include myosin density,R-d-F1%
F(imag(F)~=0) = nan;
F1(imag(F1)~=0) = nan;
  %F Results: Horizontal direction, from left to right, R is constant, d increases gradually;%
 %In the vertical direction, from top to bottom, d stays the same, and R increases%

% Analysis : P :memberane contractile pressure%%
 c1 = i./(angle1);
 y4 = transpose(y);
 y5 = repmat( y4 ,1,n+1 );
 d1  = y5 + c1;
 P = 1./d1;% ignore myosin density change,R-d-F%
 P1 = P .* m;% include myosin density,R-d-F1%
 P2 = F1 ./ y5;
 P(imag(P)~=0) = nan;
P1(imag(P1)~=0) = nan;
P2(imag(P2)~=0) = nan;
```

```
 %P Results: Horizontal direction, from left to right, R is constant, L increases gradually;%
 %In the vertical direction, from top to bottom, l stays the same, and R increases%

  %Calculation process : R-angle-F,R-angle-P %
angle = 0.1 :(pi-0.1)/n :pi;
y6 =  y.';
angle2 = y6 * angle;%x axi -angle，y axi - R%
angle3 = 1 + i./angle2;
angle4 = 1 ./ angle3;
  F2   = angle4 .* m;
   P3  = F2 ./ y5;
   F2(imag(F2)~=0) = nan;
 P3(imag(P3)~=0) = nan;


 %plot%
%%plot%%
 figure(1);
surf(x,y,F1,'FaceAlpha',0.7,'EdgeColor','none');
xlabel('d');
ylabel('R');
zlabel('F1');
%hold on
%image(x,y,F,'CDataMapping','scaled');
set(gca,'linewidth',2)

figure(2);
surf(x,y,P2,'FaceAlpha',0.7,'EdgeColor','none');
xlabel('d');
ylabel('R');
zlabel('P2');
%hold on
%image(x,y,P,'CDataMapping','scaled');
set(gca,'linewidth',2)

figure(3);
surf(x,y,F1,'FaceAlpha',0.7,'EdgeColor','none');
xlabel('d');
ylabel('R');
zlabel('F1')
%hold on
%image(x,y,F1,'CDataMapping','scaled');
hold on;
set(gca,'linewidth',2)

```

```
% define R/d=0.7 contour %
v=[0.7,0.7];
vx = repmat( x ,n+1,1 );
v1=y5./vx
[~,c]=contour(x,y,v1,v);
c.LineWidth = 4;
c.LineColor = [0 0.4492 0.4180];
hold on;

R_curved = xlsread('curved_straight.xlsx', 'Sheet1', 'A3:A55')
d_curved = xlsread('curved_straight.xlsx', 'Sheet1', 'B3:B55')
curved = scatter(d_curved,R_curved,'filled','MarkerFaceColor',[.8945 .3555 .3555]);
curved.SizeData = 120;
uistack(c);
hold on
R_straight = xlsread('curved_straight.xlsx', 'Sheet1', 'D3:D55')
d_straight = xlsread('curved_straight.xlsx', 'Sheet1', 'E3:E55')
straight = scatter(d_straight,R_straight,'filled','MarkerFaceColor',[.2578 .7109 .9297]);
straight.SizeData = 120;
uistack(c);
hold on;
R_blebb = xlsread('curved_straight.xlsx', 'Sheet1', 'G3:G55')
d_blebb = xlsread('curved_straight.xlsx', 'Sheet1', 'H3:H55')
blebb = scatter(d_blebb,R_blebb,'filled','MarkerFaceColor',[.5742 .8438 .2266]);
blebb.SizeData = 120;
uistack(c);
hold on;


figure(4);
surf(x,y,P2,'FaceAlpha',0.7,'EdgeColor','none');
xlabel('d');
ylabel('R');
zlabel('P2');
%hold on
%image(x,y,P1,'CDataMapping','scaled');
set(gca,'linewidth',2);
hold on;

[~,c]=contour(x,y,v1,v);
c.LineWidth = 4;
c.LineColor = [0 0.4492 0.4180];
hold on;
R_curved = xlsread('curved_straight.xlsx', 'Sheet1', 'A3:A55')
d_curved = xlsread('curved_straight.xlsx', 'Sheet1', 'B3:B55')
```

```
curved = scatter(d_curved,R_curved,'filled','MarkerFaceColor',[.8945 .3555 .3555]);
curved.SizeData = 120;
uistack(c);
hold on
R_straight = xlsread('curved_straight.xlsx', 'Sheet1', 'D3:D55')
d_straight = xlsread('curved_straight.xlsx', 'Sheet1', 'E3:E55')
straight = scatter(d_straight,R_straight,'filled','MarkerFaceColor',[.2578 .7109 .9297]);
straight.SizeData = 120;
uistack(c);
hold on;
R_blebb = xlsread('curved_straight.xlsx', 'Sheet1', 'G3:G55')
d_blebb = xlsread('curved_straight.xlsx', 'Sheet1', 'H3:H55')
blebb = scatter(d_blebb,R_blebb,'filled','MarkerFaceColor',[.5742 .8438 .2266]);
blebb.SizeData = 120;
uistack(c);
hold on;

figure(5);%merge F1 and P1 %
surf(angle,m,P3,'FaceAlpha',0.7,'EdgeColor','none');
xlabel('angle');
ylabel('m（myo)');
zlabel('P3')
set(gca,'linewidth',2);

 figure(6);
surf(angle,y,F2,'FaceAlpha',0.7,'EdgeColor','none');
xlabel('anlge');
ylabel('R');
zlabel('F2');
%hold on
%image(x,y,P1,'CDataMapping','scaled');
set(gca,'linewidth',2)
```

MATLAB Program4. Canny cell edge test:

The main program:

```
clc
clear
actin = imread('actin.tif');
actin = rgb2gray(actin);
actin_BW1= edge(actin,'canny');
acti_gray= double(actin_BW1);
collagen = imread('collagen.jpg');
collagen = rgb2gray(collagen);
collagen_BW1= edge(collagen,'canny');
coll_gray= double(collagen_BW1);

%actin edge%
I=acti_gray;
I(I<0) = 0;
I(I==0)=NaN;
figure(1);
x=[1:1:2048];
y=[1:1:2048];
surf(x,y,I);
view(2);

% collagen edge%
figure(2);
P = coll_gray;
P(P<1) = 0;
P(P==0)=NaN;
figure(2);
surf(x,y,P);
view(2);
```

Table S1. Primer details for qRT-PCR.

H-GAPDH	H-GAPDH-F	GGAGCGAGATCCCTCCAAAAT
	H-GAPDH-R	GGCTGTTGTCATACTTCTCATGG
H-ALP	ALP-F	GTGAACCGCAACTGGTACTC
	ALP-R	GAGCTGCGTAGCGATGTCC
H-RUNX2	RUNX2-F	TGGTTACTGTCATGGCGGGTA
	RUNX2-R	TCTCAGATCGTTGAACCTTGCTA
OCN	OCN-F	CACTCCTCGCCCTATTGGC
	OCN-R	CCCTCCTGCTTGGACACAAAG
